# Implantation in the lower half of the uterine cavity and decreased trophoblastic thickness can predict subsequent miscarriage: a prospective cohort study

**DOI:** 10.1530/RAF-23-0044

**Published:** 2023-12-13

**Authors:** Lewis Nancarrow, Nicola Tempest, Suganthi Vinayagam, Steven Lane, Andrew J Drakeley, Roy Homburg, Richard Russell, Dharani K Hapangama

**Affiliations:** 1 Centre for Women's Health Research, Department of Women's and Children's Health, Institute of Life Course and Medical Sciences, University of Liverpool, Member of Liverpool Health Partners, Liverpool, UK; 2 Hewitt Centre for Reproductive Medicine, Liverpool Women’s NHS Foundation Trust, Liverpool, UK; 3 Liverpool Women's NHS Foundation Trust, Member of Liverpool Health Partners, Liverpool, UK; 4 Department of Biostatistics, Institute of Life Course and Medical Sciences, University of Liverpool, Member of Liverpool Health Partners, University of Liverpool, UK

**Keywords:** IVF, trophoblastic thickness, pregnancy location, miscarriage, obstetric complications

## Abstract

**Abstract:**

Embryo implantation is vital for successful conception but remains to be fully understood. Trophoblast invasion is key for implantation, with anchorage and depth of placentation determined by its extent. There is a dearth of synchronous information regarding IVF, implantation site, and trophoblastic thickness (TT). Our aim was to determine whether pregnancy implantation site and TT, had an impact on outcomes of IVF pregnancies. This prospective observational study was undertaken at a tertiary referral UK fertility unit over 14 months, collecting data on implantation site and TT from three-dimensional (3D) images of the uterus following early pregnancy scan. Of the 300 women recruited, 277 (92%) had live births, 20 (7%) miscarried, 2 (0.7%) had stillbirths, and 1 (0.3%) had a termination. Significantly more pregnancies that resulted in miscarriage (7/20, 35%) were located in the lower uterine cavity when compared to ongoing pregnancies (15/277, 5%) (*P* < 0.01). TT was significantly higher in ongoing pregnancies when compared with those who miscarried (7.2 mm vs 5.5 mm; *P* < 0.01). Implantation in the lower half of the uterine cavity and decreased TT are significantly associated with an increased rate of miscarriage. Identification of those at risk should prompt increased monitoring with the aim of supporting these pregnancies.

**Lay summary:**

Implantation of an embryo in the womb is vital for a successful pregnancy. We wanted to find out whether findings on an ultrasound scan in early pregnancy had an impact on outcomes of IVF pregnancies. Three hundred women were recruited to the study, 277 (92%) had live births and unfortunately 20 (7%) had a miscarriage, 2 (0.7%) had stillbirths, and 1 (0.3%) had a termination. Many more of the pregnancies that miscarried implanted in the lower part of the womb. The thickness of the infiltration of the pregnancy into the womb was significantly higher in the ongoing pregnancies. We concluded that implantation in the lower half of the womb and reduced infiltration of the pregnancy seen on scan are associated with an increased rate of miscarriage. We propose that when we identify those at risk, we should increase monitoring, with the aim of supporting these pregnancies.

## Introduction

Embryo implantation involves a complex interaction between an embryo and the synchronised endometrium (Ochoa-Bernal & Fazleabas 2020). Although this process is vital for successful conception, it is still not fully understood (Cakmak & Taylor 2011, Bashiri *et al.* 2018). In natural conceptions, the most common site of implantation is the upper posterior portion of the uterine cavity (Kim & Kim 2017), which correlates to the highest endometrial blood flow and the most ongoing pregnancies (Jinno *et al.* 2001). The only study looking at pregnancies following *in vitro* fertilisation (IVF) found that a higher proportion of pregnancies implanted in the middle of the cavity (29.8% IVF vs 9.4% natural conception) with no difference in miscarriage rates regardless of the pregnancy location (Cavagna *et al.* 2006).

Trophoblast invasion is a key part of the implantation process and the extent of invasion determines the quality of anchorage and depth of placentation (Anin *et al.* 2004). Poor invasion increases the risk of miscarriage and other obstetric complications, such as pre-eclampsia and intra-uterine growth restriction (IUGR) (Anin *et al.* 2004). Differing information has been published relating to TT. Previously, a TT value in mm of ≥3 less than the gestation age in weeks (i.e. 4 mm TT at 7 weeks’ gestation), has been reported to be associated with an increased risk of miscarriage, implying very early placental insufficiency as the potential cause of pregnancy failure (Bajo *et al.* 2000). In contrast, a subsequent study reported that miscarriage rates were not impacted by TT (Taylor *et al.* 2019). Notably, both the studies assessed natural conceptions and did not include pregnancies achieved via assisted reproductive technology (ART). ART uses exogenous hormones, and the above studies could not account for this potential hormonal influence on the endometrium and trophoblastic invasion (Adams *et al.* 2004).

The aim of this current study was to determine whether pregnancy implantation site and TT had an impact on early and late outcomes of IVF pregnancies.

## Materials and methods

This was a prospective, observational study that recruited 300 women from August 2018 to October 2019 from a National Health Service (NHS) fertility centre. The Hewitt Fertility Centre is one of the largest reproductive medicine units in the UK, performing around 2000 IVF cycles per annum. Women were recruited, with written informed consent, following a single embryo transfer (ET) and subsequent live viable intrauterine pregnancy confirmed on a scan at 6–8 weeks’ gestation (based on day of embryo transfer). Exclusion criteria included double ET and twin pregnancy, as there may be unknown mechanisms relevant to the effects of more than one embryo on the trophoblastic invasion (Table 1) and multiple pregnancy - as could be triplet or higher order pregnancy. Uterine cavity abnormalities were also excluded due to the known effect on implantation and association with a higher miscarriage rate (Jayaprakasan *et al.* 2011, Simon & Laufer 2012) (Table 1).

**Table 1:** Inclusion and exclusion criteria.

Inclusion criteria
Single ET
Single viable intrauterine pregnancy
Able to provide informed consent
Exclusion criteria
≥2 embryos transferred
Multiple pregnancy
Uterine cavity abnormalities (e.g. submucosal fibroids, septate uteri)
Unable or unwilling to provide informed consent

Ultrasound scans were obtained using a General Electric Volusen E8 ultrasound machine and a 3D/4D RIC5-9-D transvaginal probe (GE Medical Systems Kretztechnik GmbH & Co).

Images were stored and a single, experienced operator blinded to the outcomes retrospectively calculated all measurements.

**Figure 1:**
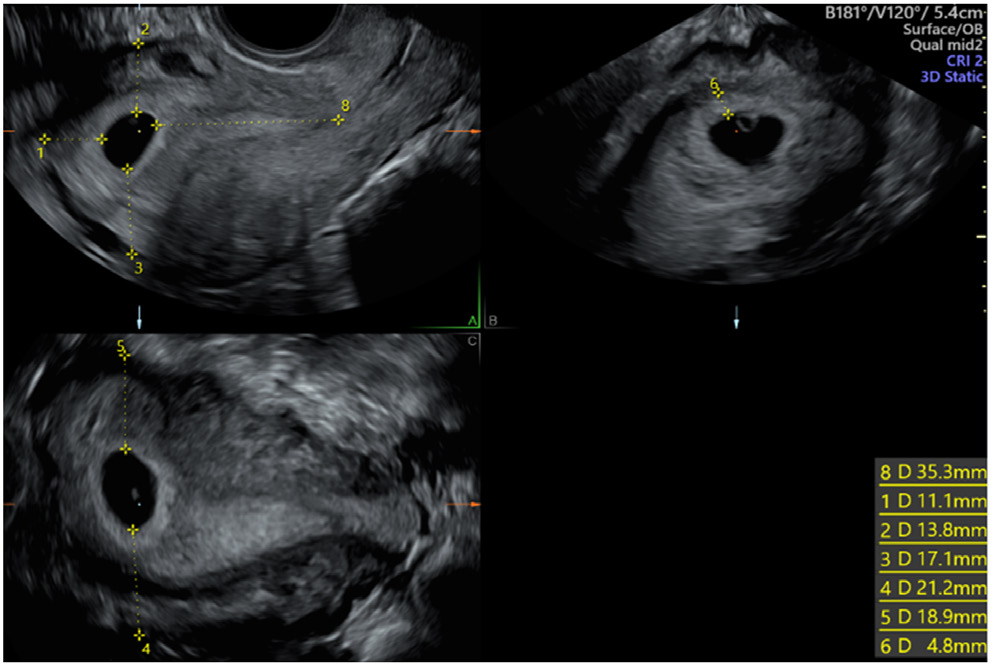
Measurements acquired: 1 – Distance from fundus. 2 – Distance from anterior uterine wall. 3 – Distance from posterior uterine wall. 4 – Distance from right uterine wall. 5 – Distance from left uterine wall. 6 – Trophoblastic thickness. 8 – Distance from internal cervical os.

Implantation site was determined by measuring the minimum distance from the gestation sac to the anterior and posterior of the uterus, the uterine fundus, the lateral edges of the uterus, and the internal cervical os (Fig. 1). For example, If the distance from the internal cervical os was 25 mm and it was 20 mm from the fundus then the pregnancy was deemed to be in the upper portion of the uterus. If the measurements were the same then the gestation sac was deemed to be in the middle of the cavity in that plane. TT was measured in the anterior–posterior diameter in the anterior aspect of the uterus.

Demographic variables collected included, woman’s age, BMI, type of infertility, type of ET (fresh or frozen), and embryo quality (Table 2).

**Table 2:** Demographic data and baseline characteristics for all women with a live birth or miscarriage. Data are presented as mean ± S.D. or *n* (%).

	Live birth (*n* = 277)	Miscarriage (*n* = 20)	*P*
Mean age (years)	33.5 (± 3.89)	35.9 (± 3.35)	**<0.01**
Mean BMI (kg/m^2^)	24.6 (± 3.41)	25.3 (± 2.54)	0.38
Type of infertility			0.77
Primary	120 (43)	10 (50)	
Secondary	157 (57)	10 (50)	
Type of ET			0.53
Fresh	107 (39)	5 (25)	
Frozen	170 (61)	15 (75)	
Embryo quality (*n* = 270*)			0.84
Good	190 (70)	16 (80)	
Average	64 (24)	4 (20)	
Poor	16 (6)	0 (0)	

Bold values indicate statistical significance. *Seven of the embryos were transferred on day 3; therefore, they were unable to be graded according to the Gardner and Schoolcraft grading system (Gardner 1999).

The primary outcome was miscarriage rate (pregnancy loss <24 weeks’ gestation). Secondary outcomes included live birth rate (LBR), stillbirth (baby born >24 weeks’ gestation without a heartbeat), termination of pregnancy (TOP), obstetric complications (gestational diabetes mellitus (GDM), pre-eclampsia, SGA, placenta praevia, and placenta accreta), birth weight, and gestational age at delivery. Numbers with stillbirths and TOPs were too small and therefore not included in the statistical analysis.

Using the population-based growth chart (Kiserud *et al.* 2017), birth weights were classed as either SGA (SGA, growth <10th centile (Sovio *et al.* 2015)), appropriate for gestational age (AGA, growth between 10th and 90th centile), or large for gestational age (LGA, growth >90th centile (Modzelewski *et al.* 2021)).

Outcome data were obtained via local electronic hospital records (MEDITECH) or via direct telephone communication with the patients. Data were uploaded onto Microsoft Excel (Microsoft Excel 2019) prior to analysis.

### Statistical analysis

Data were migrated from Microsoft Excel to the Statistical Package for the Social Sciences Statistics (SPSS, version 26; IBM Corporation) for analysis. Continuous data were analysed using Student’s *t*-test, whilst categorical data were analysed using the *χ*^2^ test. When means of more than two groups were compared, one-way ANOVA test was used. Significance was achieved when the two-sided *P*-value was <0.05.

The study was approved by the Cheshire and East Midlands – Leicester Central Research Ethics Committee (REC 16/EM/0392), and all women gave a written consent.

## Results

Three hundred women were recruited between 6 weeks and 3 days gestation to 8 weeks and 6 days gestation. Of the 300 women recruited, 277 (92.3%) achieved a live birth, 20 (6.7%) had a miscarriage, 2 (0.7%) had stillbirths, and 1 (0.3%) had a TOP for trisomy 21. The data from the 297 women who had live births or miscarriages were included in the analysis.

The group of women who subsequently had a miscarriage were significantly older than the women who had a live birth (*P* < 0.01) (Table 2). BMI, types of infertility, ET, and embryo quality were similar between the two groups of women (Table 2).

### Primary outcome

Women with a pregnancy located in the lower half of the uterus were more likely to miscarry compared with those women who had a live birth (35% vs 5.5%, *P* < 0.01) (Table 3). There was a poor association between distance from internal os and pregnancy outcomes with an area under the curve analysis of 0.64 (95% CI: 0.48–0.80).

**Table 3:** Pregnancy location and outcomes. Data are presented as *n* (%).

Pregnancy location	Live birth (*n* = 277)	Miscarriage (*n* = 20)	*P*
Upper-lower			**<0.01**
Upper	261 (94)	13 (65)	
Middle	1 (0.5)	0	
Lower	15 (5.5)	7 (35)	
Right-left			0.40
Right	148 (53)	7 (35)	
Middle	7 (3)	0	
Left	122 (44)	13 (65)	
Anterior-posterior			0.84
Anterior	128 (46)	12 (60)	
Middle	7 (3)	0	
Posterior	142 (51)	8 (40)	

Bold value indicates statistical significance.

**Figure 2:**
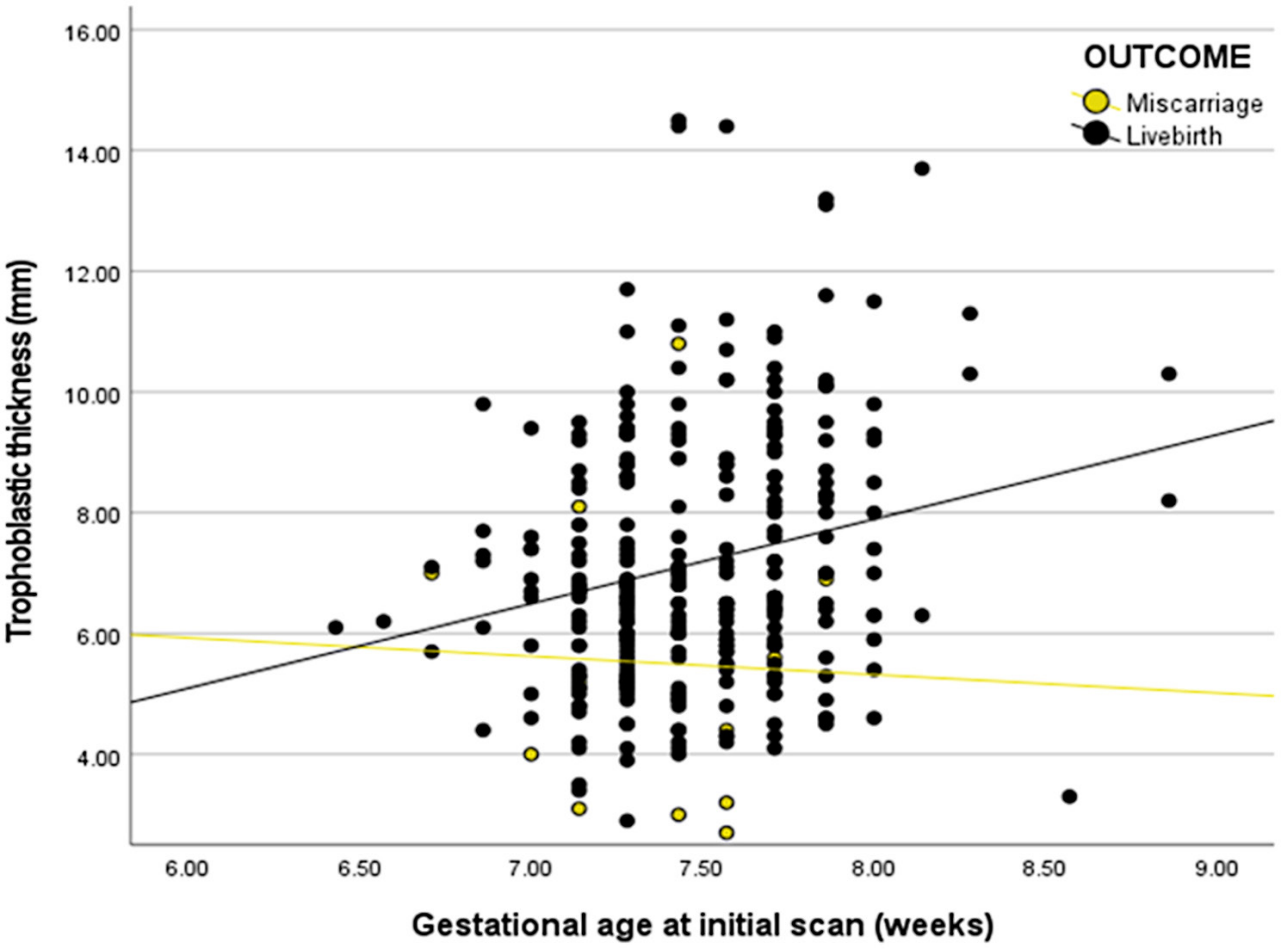
Trophoblastic thickness and relationship to pregnancy outcome.

TT was significantly more in those women who had a live birth compared to those who miscarried (7.2 mm ± 2.1 vs 5.5 mm ±2.0; *P* < 0.001) (Fig. 2). On binary logistic regression analysis, TT, gestational age at scan, and pregnancy outcome remained significant (*P* < 0.01).

### Secondary outcomes

#### Obstetric complications

Of the 277 live births, 59 (27.1%) had obstetric complications (some pregnancies suffered more than one complication). When considering all complications, those pregnancies located in the middle, in the transverse plane, rather than in the right or left of the cavity, were more likely to have obstetric complications, although the total number of pregnancies in the middle is small (Table 4).

**Table 4:** Pregnancy location and obstetric complications. Data are presented as *n* (%).

	No complications (*n* = 218)	Obstetric complications (*n* = 59)	*P*
Anterior–posterior			0.68
	Anterior	98 (45)		30 (51)
	Middle	6 (3)		1 (2)
Posterior	114 (52)	28 (47)	
Upper–lower			0.87
	Upper	205 (94)		56 (95)
	Middle	1 (0.5)		0 (0)
Lower	12 (5.5)	3 (5)

Left–right			**0.03**
	Left	101 (46)		21 (35)
	Middle	3 (2)		4 (7)
Right	114 (52)	34 (58)	

Bold values indicate statistical significance.

Considering specific complications, the only significant association was those in the middle of the uterine cavity in the transverse plane were more likely to develop GDM and pre-eclampsia (Table 5).

**Table 5:** Subgroup analysis of pregnancies located on the left, middle, or right with obstetric complications*. Data are presented as mean ± s.d.or *n* (%).

	Left (*n* = 21)	Middle (*n* = 4)	Right (*n* = 34)	*P*
Mean age (years)	33.9 (±4.10)	33.5 (±3.11)	33.3 (±3.52)	0.86
Mean BMI (kg/m^2^)	26.12 (±3.78)	24.91 (±5.10)	25.08 (±2.99)	0.54
GDM	7 (33)	4 (100)	14 (41)	**<0.001**
Pre-eclampsia	3 (14)	2 (50)	8 (24)	**0.005**
SGA	3 (14)	0 (0)	4 (12)	0.90
Placenta praevia	4 (19)	0 (0)	2 (6)	0.51
Placenta accreta	0 (0)	0 (0)	2 (6)	0.42
LGA	5 (24)	1 (25)	8 (24)	0.47

Bold values indicate statistical significance.

*Some pregnancies had more than one complication.

Babies classed as LGA had a significantly increased TT (*P* < 0.01), but no other statistical findings were seen with regard to TT and obstetric complications (Table 6).

**Table 6:** Trophoblastic thickness and obstetric complications. Data are presented as mean ± s.d. or *n* (%).

	Number (277)	Mean TT (mm)	Obstetric complication vs no complication *P*
Obstetric complication			0.89
No	218 (79)	7.15 (±2.08)	
Yes	59 (21)	7.20 (±2.23)	
GDM	25 (9)	6.85 (±2.04)	0.44
Pre-eclampsia	13 (4)	6.61 (±1.61)	0.33
SGA	7 (3)	6.41 (±2.14)	0.34
Placenta praevia	6 (2)	7.82 (±3.13)	0.45
Placenta accreta	2 (1)	6.05 (±1.91)	0.46
LGA	14 (5)	8.93 (±1.73)	**<0.01**

Bold value indicates statistical significance.

#### Birth weight

There was no significant association between pregnancy location and birth weight or TT and birth weight.

#### Gestational age at delivery

Pregnancies located in the middle of the uterine cavity were more likely to be delivered earlier than those pregnancies located at either the left or right side of the uterine cavity (*P* < 0.01) (Table 7).

**Table 7:** Pregnancy site location and gestational age. Data are presented as *n* (%).

	Number (total *n* = 277)	Mean gestational age (weeks)	*P*
Left-right			<0.01
Left	122 (44)	39.0	
Middle	7 (3)	36.8	

Right	148 (53)	39.0

It should be noted that this finding was specific only to the transverse plane but a similar effect was not observed in the sagittal or coronal planes.

## Discussion

In agreement with previous natural conception studies that examined the relevance of implantation site to pregnancy outcomes, we have demonstrated an increased miscarriage rate in IVF pregnancies that are located in the lower portion of the uterine cavity (Kinoshita 1994, Minami *et al.* 2003). Our findings are also in agreement that a decreased TT is more likely to lead to miscarriage in IVF pregnancies (Bajo *et al.* 2000).

We demonstrate that information regarding pregnancy location and TT on an early ultrasound scan gives useful information about IVF pregnancy outcomes. As the endometrial perfusion is less in the lower portion of the uterus when compared to the fundus (Jinno *et al.* 2001, Minami *et al.* 2003, Jansen *et al.* 2020), a pregnancy establishing in the lower part of the cavity may be less able to support the requirements of an advanced pregnancy. Although a previous study reported no association this could have been confounded by the small number of participants (*n* = 63) and the inclusion of multiple pregnancies (Cavagna *et al.* 2006).

For the first time we considered the intracavity location of the pregnancy as a predictive marker of obstetric complications. When a pregnancy was located in the middle of the cavity, in the transverse plane, there was a significantly higher incidence of total occurrence of all obstetric complications examined when compared with those pregnancies located more to the right or left side of the cavity. However, when the complications were assessed individually, this significant difference was lost, except for those who developed GDM and pre-eclampsia. Similarly, those pregnancies in the middle of the uterus were more likely to be delivered earlier than those on the right or left. We are uncertain as to the clinical significance of these findings, but further studies are required to see if these results can be replicated.

All available previous data regarding TT have been reported using naturally conceived pregnancies; therefore, this may not be relevant to those conceived following IVF (Bajo *et al.* 2000, Taylor *et al.* 2019, Hanchard *et al.* 2020). Our data from pregnancies conceived via IVF are in agreement with the first paper published in relation to TT and pregnancy outcomes, including that those with decreased TT were more likely to miscarry (Bajo *et al.* 2000), but in conflict with the more recent study assessing TT and miscarriage (Taylor *et al.* 2019). Our study was in agreement with the previous study reporting that TT was not altered in pregnancies complicated with hypertension (Hanchard *et al.* 2020).

Our study demonstrated a significant difference in TT of AGA versus LGA babies, but no difference between the TT of SGA versus LGA, probably secondary to the small number of pregnancies with an SGA baby. A future study containing a larger cohort of patients would be required to clarify these findings. Interestingly, except for placenta praevia, women with all other late obstetrics complications studied, such as GDM, pre-eclampsia, SGA, and placenta accreta had apparently thinner TT when compared with the control group without complications, but this did not reach statistical significance. A decreased TT may represent suboptimal placentation from a pathophysiological perspective, and most of these complications have a relevance to suboptimal trophoblastic invasion, e.g. pre-eclampsia and SGA (Scifres & Nelson 2009, Chaiworapongsa *et al.* 2014, Gamage *et al.* 2020).

This is the first study to consider the association of pregnancy location and TT measured by 3D ultrasound scanning with both early and late pregnancy complications in IVF pregnancies. Observer bias was reduced by the fact that the measurements were made by a single, experienced practitioner using the same ultrasound machine for recording all measurements of pregnancy location and TT (Axell *et al.* 2012). 3D imaging has previously been shown to improve inter-observer reproducibility in comparison to 2D imaging. It also allows for postprocessing reviews of the images, which would not be obtainable with 2D imaging (Coyne *et al.* 2008, Pascual *et al.* 2011). Despite finding statistically significant differences for the primary outcome (miscarriage rates), this study was not sufficiently powered to detect significant associations of ultrasound features with the other secondary outcomes. 3D ultrasound is not routinely used in early pregnancy scanning since it requires more advanced and costly ultrasound machines and training. This potentially limits the translatability of our research into routine clinical practice. Determining the pregnancy site location was based on scans between 6 and 8 weeks in gestation. Whilst pregnancy location was determined using this method, in future it may be more beneficial to consider an earlier scan in the pregnancy to determine the true implantation site.

Future studies to confirm our results should include a larger cohort of patients, including other ultrasound markers such as trophoblastic volume (TV), mean gestational sac diameter, fetal heart rate (FHR), and mean uterine artery pulsatility index (UAPI). An appropriate control group of naturally conceived pregnancies and the use of other biochemical markers, as well as facilitating studies into trophoblast invasion, could lead to the development of more effective therapeutic strategies, promoting optimal trophoblast invasion to minimise the burden and impact of early and late pregnancy complications in IVF pregnancies.

## Conclusions

This novel study has shown that pregnancy location in the lower half of the uterus and decreased TT are more likely to result in miscarriage in IVF pregnancies. These findings along with other ultrasound markers such as TV, mean UAPI, and FHR can be used to develop an algorithm to better counsel and manage those at risk of early pregnancy loss and obstetric complications. Identification of those at risk may also prompt increased monitoring during pregnancy, such as attendance at high-risk antenatal clinics and extra growth scans, with the aim of reducing both maternal and neonatal morbidity. Further basic science studies examining the effect of sheer mechanical pressure and other location related properties within the uterine cavity may explain the observed differences affecting embryo implantation.

## Declaration of interest

The authors declare that there is no conflict of interest that could be perceived as prejudicing the impartiality of the study reported.

## Funding

This work was funded by the Hewitt Fertility Centre.

## Author contribution statement

Conceptualisation and ethical approval: RH and RR; LN and SV collected the data, NT, SL, LN, and DKH analysed and interpreted the data and wrote the first draft of the manuscript. All authors read and agreed to the final version of the manuscript.
